# Risk assessment and predator learning in a changing world: understanding the impacts of coral reef degradation

**DOI:** 10.1038/srep32542

**Published:** 2016-09-09

**Authors:** Douglas P. Chivers, Mark I. McCormick, Bridie J. M. Allan, Maud C. O. Ferrari

**Affiliations:** 1 Department of Biology, University of Saskatchewan, SK, Canada; 2 ARC Centre of Excellence for Coral Reef Studies, and College of Marine & Environmental Sciences, James Cook University, Townsville, QLD, Australia; 3 Department of Biomedical Sciences, WCVM, University of Saskatchewan, SK, Canada

## Abstract

Habitat degradation is among the top drivers of the loss of global biodiversity. This problem is particularly acute in coral reef system. Here we investigated whether coral degradation influences predator risk assessment and learning for damselfish. When in a live coral environment, Ambon damselfish were able to learn the identity of an unknown predator upon exposure to damselfish alarm cues combined with predator odour and were able to socially transmit this learned recognition to naïve conspecifics. However, in the presence of dead coral water, damselfish failed to learn to recognize the predator through alarm cue conditioning and hence could not transmit the information socially. Unlike alarm cues of Ambon damselfish that appear to be rendered unusable in degraded coral habitats, alarm cues of Nagasaki damselfish remain viable in this same environment. Nagasaki damselfish were able to learn predators through conditioning with alarm cues in degraded habitats and subsequently transmit the information socially to Ambon damselfish. Predator-prey dynamics may be profoundly affected as habitat degradation proceeds; the success of one species that appears to have compromised predation assessment and learning, may find itself reliant on other species that are seemingly unaffected by the same degree of habitat degradation.

Climate change, together with crown of thorn outbreaks, overfishing and pollution, is a major driver of coral reef degradation^1^,^2^,^3^. In the last two decades, increases in the frequency and severity of storms, as well as episodes of coral bleaching and disease have greatly impacted coral reefs globally^4^,^5^. As corals die, they lose their outer tissue and may be overgrown by algae. Over time, coral skeletons break down leaving a rubble-dominated habitat^6^. While this progression of habitat change is natural, the speed at which it is occurring is unprecedented^7^. Indeed, estimates suggest that 60% of reefs are facing extinction and may be lost by 2030^8^. Even the Great Barrier Reef, often regarded as one of the most pristine reefs in the world, has seen considerable degradation^9^.

For fish species living on coral reefs, the death of a coral patch and subsequent transition to a rubble dominated habitat represents a major form of ecosystem degradation. Species must either move to less affected habitat patches, or be faced with trying to survive in the degraded environment. Many species use live coral as a nursery habitat^10^,^11^,^12^ and these juveniles have a low willingness to migrate even when faced with higher quality habitat nearby^13^. These juveniles are also the most vulnerable due to their small size and naiveté to reef predators.

Our current research focuses on how fish deal with the critical issue of risk assessment within the context of this major habitat change. Many aquatic organisms, including fishes, use chemical cues to mediate their risk of predation^14^,^15^. Alarm cues released from injured conspecifics provide animals with immediate knowledge that a predator was recently feeding in the area, and unsurprisingly, prey that have been ‘warned’ with alarm cues have higher survival than those that were ‘not warned’^16^. Perhaps even more important, alarm cues mediate learned recognition of predators^17^. When prey fish detect alarm cues paired with the sight, sound or odour of an unknown predator, they learn the identity of the predator through a Pavlovian-like conditioning paradigm^14^,^15^. This mode of learning is crucial as it allows prey to learn about novel predators without being directly attacked. These experienced individuals are then able to socially transmit the information about the learned predator to other nearby naïve conspecifics^18^,^19^,^20^. Social learning, or cultural transmission, is a mode of learning found in a wide diversity of taxa both within and across-species and in a number of ecological contexts, such as foraging and mate choice^20^. Social learning of predator identity allows for the rapid transmission of predator-related information within a population^21^ and provides documented survival benefits in the wild^22^.

A few studies have recently suggested that risk assessment by Ambon damselfish *(Pomacentrus amboinensis)* may be compromised in degraded habitats^23^,^24^,^25^. Indeed, these damselfish have a limited ability to use alarm cues for risk assessment when their alarm cues have been mixed with even small amounts of water from degraded coral habitats. However, whether this response reflects a lack of detection of the cues or a lack of motivation to respond to the cues is uncertain. The lack of an overt behavioural response does not necessarily mean that the prey cannot incorporate this information into their risk decision-making algorithm^26^. Whilst the vast majority of prey animals show a somewhat limited ability to recognize predators in the absence of experience^17^, there is some evidence that some fish may respond to predator cues without experience^27^,^28^. This includes studies on damselfishes, which have been found to sometimes avoid predator odours at or near the time of settlement^29^,^30^,^31^. This result was later explained by the transient neophobic tendencies of juveniles living in high-risk environment^32^,^33^. Regardless of whether a species has innate recognition or not, learning provides a great opportunity to enhance and fine-tune responses to predators that are present in the environment.

In the present study, we asked whether coral degradation altered the ability of the *P. amboinensis* to learn risk and if so, could they still learn risk via social learning from other species whose learning was not comprised in a degraded habitat. First, we tried to teach *P. amboinensis* the identity of an unknown predator with alarm cues and then tested whether they could transmit the information to naïve conspecifics through social learning. Tests were conducted in both healthy coral water and in degraded coral water. We predicted that if *P. amboinensis* did not respond to alarm cues in degraded coral habitats, then they should fail to learn the identity of the predator during the training procedure, and hence not be able to socially transmit this information to naïve observers. In a second experiment, we investigated whether *P. amboinensis* could compensate for the effect of degraded coral on their risk assessment by learning risk from a close rubble-dwelling heterospecific, the Nagasaki damselfish (*Pomacentrus nagasakiensis*).

## Methods

### Test species

The focal species for the study, *P. amboinensis*, is a common species on Indo-Pacific reefs, which commonly settles to similar habitats to the heterospecific, *P. nagasakiensis* (Kerrigan 1996). Newly metamorphosed, settlement-stage juvenile *P. amboinensis* and *P. nagasakiensis* were caught overnight using light traps moored in open water around Lizard Island (14′40° S, 145′28° E), in the northern Great Barrier Reef, Australia in November 2015. The juveniles, sorted by species, were then placed in 20-L flow-through holding tanks and fed three times a day with brine shrimp (*Artemia* nauplii). Because the fish were caught before they settled on the reef, they are naïve to the suite of predators that awaits them on the reefs. Eight dottybacks, *Pseudochromis fuscus*, (range: 11.3–14.1 cm total length) were caught on SCUBA using hand nets and clove oil and were used as the predatory cue. These are small but voracious predators that prey on recently settled fishes^34^. Fish were housed individually in 1-l mesh containers that were floating inside of 20-l plastic tubs. Two fish were kept in each tub. Separating the fish ensured that they could not fight. Water flowed into the tubs at a rate of approximately 1 l per minute. The predators were fed daily with squid. Experiments began after the fish were held in the laboratory for a minimum of one week.

### Experimental setup

Our social learning experiments followed three steps: (1) obtain predator-naïve and predator-experienced tutors; (2) pair a naïve observer with a tutor (naïve or experienced) and expose them to predator cues, (3) test the observer alone for their response to the predator cue to determine whether they had learnt from the tutor. Each of these steps is described below, and an overview of experimental manipulation summarized afterwards. All experimental methods were carried out in accordance with relevant guidelines and regulations; all protocols were approved by the James Cook University Animal Ethics Committee (Approval numbers: A2080 and A2005).

#### Training tutors

Fish to be used as tutors were split into two groups, a predator-experienced group being trained to recognize the odour of a predator as threatening and a predator-naïve group undergoing a false training. Fish were placed individually in 5-L tanks containing a sandy substrate, a plastic branching coral model and flowing seawater, and were left to acclimate for 3 h. All tanks were visually isolated on all four sides, to avoid information transfer from adjacent tanks and to decrease disturbance from the experimenters. The predator-experienced tutors were exposed to 15 mL of predator odour paired with 5 mL of injured conspecific cues. These cues elicit innate dramatic antipredator responses and mediate learned predator recognition in a wide variety of aquatic species^15^. Injured cues were prepared by sacrificing donor conspecifics via cold shock, and making five vertical cuts on the side of each flank of two fish. The donor were fish of the same size as used in the experiment, therefore each cut was approximately 7 mm in length. The donors were then rinsed with 10 mL of seawater and the solution was used immediately. The 5 mL to be injected led to a final concentration of ~2 cuts/L in the tank, a concentration known to elicit overt antipredator responses in our test species^35^. The predator-naïve tutors were exposed to 15 mL of predator odour, paired with 5 mL of seawater. The predator odour was made from soaking two live dottybacks in 3 L of aerated seawater for 1 h. After this training, the tutors were left undisturbed for 1 h, and were then transferred to an identical 3-L tank containing clean aerated seawater.

#### Training of naïve observers by tutors

Each tutor was paired with a randomly chosen naïve observer. To distinguish between tutors and observers, all the observers in our experiments were marked, five days prior to being used, with a small elastomer tag on the dorsal part of their body (as per Hoey and McCormick^36^). The pair was left to acclimate for 3 h. Each tank then received 2 mL of a food solution containing ~100 *Artemia*/mL. After 3 min, we slowly injected 15 mL of predator odour in the tanks using a 1.5 meter long plastic tube. The stimuli were flushed into the tanks with 20 ml of seawater. Here, the prediction is that predator-naïve tutors will not respond to the introduced cue and would continue to feed, providing no information about risk to the observer. Predator-experienced tutors, on the other hand, would display a marked antipredator response, by reducing feeding and activity. This change in behaviour, in association with the novel cue, should indicate to the naïve observer that the cue is threatening^37^. After the cue introduction, the pair was left undisturbed for 1 h. The observer was then placed, alone, into a similar 5-L tank. These tanks were equipped with a 1.5 m long injection hose used to introduce food and cue during observations. The tanks were visually isolated on three sides only. The fourth side, used for observation, had a 4 × 4 cm grid drawn on it, used to quantify activity (see below).

#### Testing of the observer

The following day, the observers were tested for their response to the odour of the predator. Three min prior to the start of the observation, we injected 2 mL of food solution in the tank. We injected another 2 mL of food and started the 3-min pre-stimulus observation. We recorded two variables: number of feeding strikes, regardless of whether the fish was successful or not, and the number of lines crossed, as a measure of swimming activity, using the grid drawn on the tank. We then injected 15 mL of predator odour, 2 mL of food and started the 3-min post-stimulus observation, during which we recorded the same variables. The change in behaviour from the pre-stimulus to the post-stimulus period indicates the response of the fish to the stimulus. Typical antipredator responses include decreased foraging and activity^38^. At the end of the testing, all observers and their tutors were measured.

### Experiment 1: Can *P. amboinensis* transfer information socially in degraded habitat?

In this experiment, we tested whether *P. amboinensis* could socially learn in degraded habitats. We carried out a 2 × 3 experimental design. Naïve *P. amboinensis* observers were paired with either predator-naïve or predator-experienced tutors (predator experience). We also manipulated the habitat in which training and testing took place. In the first treatment (live-healthy coral), all three phases of the social learning experiment took place in seawater that had passed over live coral. In the second treatment (dead-degraded coral), all three phases of the social learning experiment took place in seawater that had passed over dead-degraded coral. These two scenarios represent situations that would naturally occur in the wild. A third group was added to decipher the cause of potential differences between the first two groups. In this third treatment, the first phase took place in seawater that had passed over live coral, while the last two phases took place in dead-degraded coral water. This treatment was designated live/dead. We tested 10–12 fish in each predator-naïve treatment and 19–21 fish in each of the predator-experienced treatments. The asymmetry reflects the lower sample size required for the control groups. We have conducted several similar learning studies and were confident not to expect social learning when the tutors were not trained to recognize the predator^37^,^39^,^40^.

**Figure 1:**
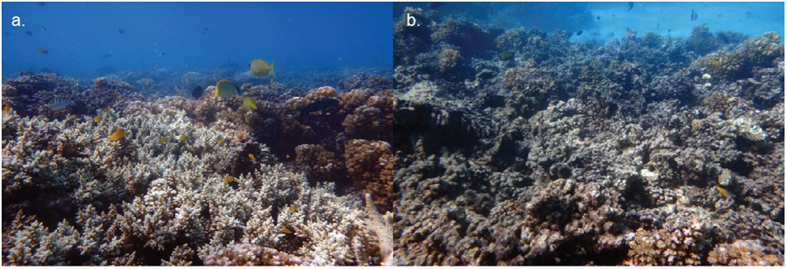
(**a**) Area of healthy reef with good coverage of live hard coral; (**b**) adjacent area of degrading coral with a high percentage of dead, algal-covered coral.

The type of seawater entering experimental tanks was controlled by header tanks. We used eight different header tanks, each containing a 60-cm diameter piece of either live-healthy or dead-degraded *Pocillopora damicornis*, a hard bushy coral common at our field site (Fig. 1). Fresh seawater flowed through the header tanks into the 5-L tanks at a rate of 1 L/min. The outflow was then split into five tanks, with a resulting flow of about ~1 L/5 min, which represents a complete turnover of tank water in 25 min. Both types of corals were replaced every two days.

### Experiment 2: Can *P. amboinensis* learn socially in degraded habitat from heterospecifics?

This experiment followed a 2 × 2 × 2 design, whereby the tutor was either naïve or experienced with the predator (experience), all phases of the experiment took place in either live or dead coral water (habitat), and the tutor was either a conspecific *P. amboinensis* or a heterospecific *P. nagasakiensis* (tutor).

### Statistical analysis

For both experiments, tutors and observers were randomly paired. However, we tested for any potential bias in size pairing between tutors and observers among treatment groups using a 3-way ANOVA.

The two behaviours recorded (foraging and activity) were analysed together using a MANOVA approach. We first tested for any behavioural bias of observers by comparing their baseline (pre-stimulus) activity and foraging levels across treatment groups. We then computed a proportion change in behaviour ((post-pre)/pre) and used these variables in our subsequent analyses.

For experiment 1, we performed a 2-way MANOVA testing the effect of tutor experience (predator-experienced vs. predator-naïve) and coral habitat (live vs. dead vs. live/dead). Data were split by habitat to investigate the nature of interactions. Differences among treatments were investigated with Tukey post-hoc tests.

For experiment 2, we ran a 3-way MANOVA, testing the effect of tutor experience (predator-naïve vs. predator-experienced), tutor species (*P. amboinensis* vs. *P. nagasakiensis*) and coral habitat (live vs. dead). Data were split by habitat to investigate the nature of any interactions.

All data met parametric assumptions (Komolgorov-Smirnoff tests for normality and Levene’s tests for homoscedasticity: all P > 0.05). Analyses were performed with SPSS 23.

## Results

### Experiment 1

**Figure 2:**
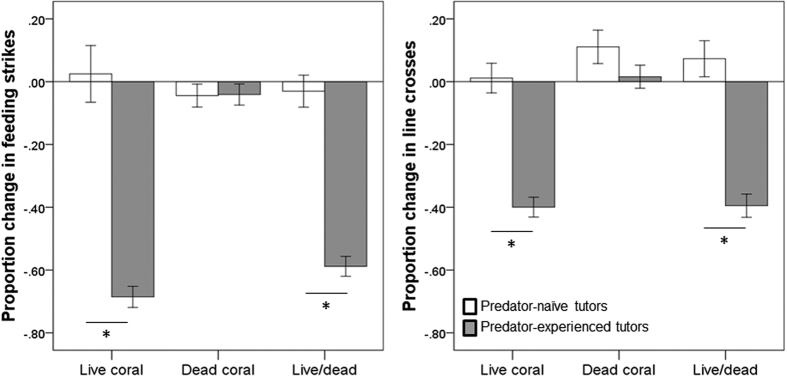
Mean (±SE) proportion change in feeding strikes (left) and line crosses (right) for observers paired with predator-naïve tutors (empty bars) or predator-experienced tutors (solid bars). The three-phase training sequence took place in either live coral water (live coral) or dead coral water (dead coral). For the last group, the tutors were trained in live coral, but the training and testing of observer took place in dead coral water (live/dead). *Indicates significant differences between treatments at P < 0.001.

All observers displayed similar baseline behaviour regardless of the experience of their tutors (Pillai’s Trace: F_2,86_ = 1.4, P = 0.2), and regardless of habitat type (Pillai’s Trace: F_4,174_ = 0.6, P = 0.6; Tutor experience x Habitat: F_4,174_ = 1.5, P = 0.2), indicating no bias among groups. Fish displayed on average 71 ± 22 (mean ± SD) line crosses and 46 ± 16 feeding strikes during the prestimulus period. Their behavioural response to the predator odour were affected by an interaction between tutor experience and habitat (F_4,174_ = 16.1, P < 0.001, Fig. 2). In water that had passed over dead coral, tutor experience did not affect the response of the fish to the predator odour (F_2,29_ = 1.2, P = 0.3), indicating the observers failed to recognize the predator odour as threatening. In live coral water, however, observers paired with experienced tutors responded to the predator odour with a strong antipredator response (F_2,28_ = 77.6, P < 0.001). When the tutors were trained in live coral water but the observer sequence took place in dead coral water (live/dead treatment), we also found that observers paired with experienced tutors displayed a strong response to the predator compared to those paired with naïve tutors (F_2,27_ = 59.8, P < 0.001). In fact, the responses of the observers from the live and live/dead treatments were of similar intensities (Tukey post-hoc comparison: P > 0.9). Size differences between tutors and observers did not differ among treatments (2 × 3 ANOVA: all P > 0.2).

### Experiment 2

**Figure 3:**
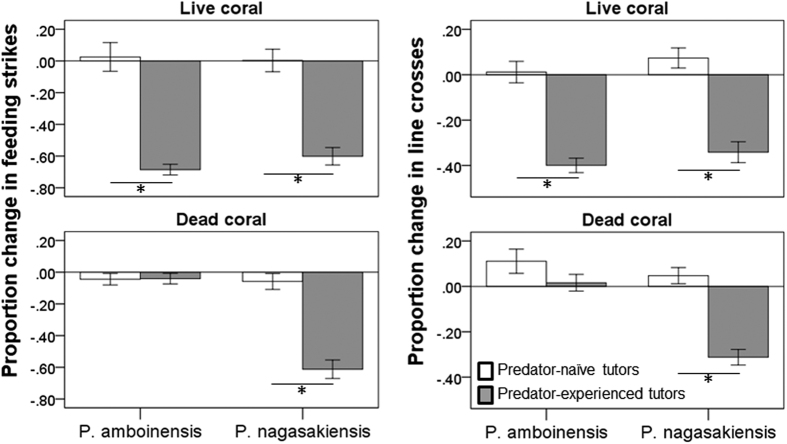
Mean (±SE) proportion change in feeding strikes (left) and line crosses (right) for *P. amboinensis* observers paired with predator-naïve tutors (empty bars) or predator-experienced tutors (solid bars). Tests were conducted in live coral water or dead coral water and the tutors were either *P. amboinensis* or *P. nagasakiensis*. *Indicates significant differences between treatments at P < 0.001.

We failed to find a significant effect of any factors or interactions on the pre-stimulus behaviour of the fish (all P > 0.2), indicating that all fish displayed the same baseline behaviour (line crosses: 59 ± 24, feeding strikes: 45 ± 16). When we looked at the change in behaviour in response to predator odour, we found it to be affected by a 3-way interaction among tutor experience, tutor species and habitat (Pillai’s Trace: F_2,115_ = 10.5, P < 0.001, Fig. 3). In the live coral environment, the observers responded stronger to the predator when paired with an experienced tutor than with an naïve tutor (F_2,58_ = 91.9, P < 0.001), but the responses were not affected by tutor species (F_2,58_ = 0.9, P = 0.4) or any interaction between the tutor species and tutor experience (F_2,58_ = 0.4, P = 0.7). This indicated that fish learned to recognize the predator as threatening when paired with a predator-experienced tutor, regardless of the tutor species. In dead coral environments, however, the response of the fish was affected by an interaction between experience and species (F_2,56_ = 20.1, P < 0.001). Indeed, observers could learn to recognize the predator when the tutor was *P. nagasakiensis* (experience: P < 0.001), but failed when the tutor was *P. amboinensis* (experience: P = 0.3). Size difference between tutor and observers was affected by tutor species (2 × 2 × 3 ANOVA, P < 0.001), an expected result given that *P. nagasakiensis* are larger than *P. amboinensis*. None of the other factors affected the size difference (all P > 0.1).

## Discussion

The results of our study demonstrate that risk assessment and learning of predators can be dramatically compromised in degraded habitats for fish that most commonly associate with live coral environments. In our first experiment, we showed that in healthy coral water, *P. ambonensis* tutors that were trained with alarm cues transmitted the recognition of predator odour to naïve conspecific observers. In contrast, naïve conspecific observers did not learn when they were paired with predator-inexperienced tutors. These results demonstrate both learned recognition of predators through conditioning with alarm cues and the subsequent social transmission of this recognition to naïve conspecifics, and as such, supports previous work on learning mechanisms in coral reef fishes^19^,^39^,^40^. However, the striking results are that damselfish failed to learn when similar procedures were undertaken in degraded coral water. These results on their own did not allow us to determine whether the failure stemmed from a failure from the tutor (step 1) or one from the observer (steps 2 and 3). For that reason, we included a treatment whereby the alarm cue training (step 1) took part in live coral seawater and the social learning component of the experiment (steps 2 and 3) took place in degraded habitat water. The results from our live/dead treatment provided clear evidence for a failure of step 1 in the dead/degraded coral treatment. Steps 2 and 3 appeared unaffected by the degraded coral environment. Indeed, if the tutor was trained with alarm cues in healthy coral water, it was able to act as a tutor in degraded coral water, and other fish were able to learn from observing them displaying antipredator responses in degraded coral water. The intensity of the learned response displayed by observers from the ‘live/dead’ treatment was in fact similar to that displayed by fish tested in healthy coral water. These results indicate that social leaning – i.e., the transmission of information from knowledgeable conspecifics to naïve observers, still operate under degraded habitats without waning of the response intensity. However, the failure of step 1 implied that the failure of alarm cues, which play a major role in a number of trait-mediated indirect effects, may result in a lower occurrence of social learning in the group due to a decrease in individuals serving as information sources.

In our second experiment, we investigated the effects of habitat degradation on risk assessment and learning in a cross-species context, asking whether the source of information could come from heterospecifics that are less susceptible to coral degradation. Just like experiment 1, we showed that *P. amboinensis* could learn predators and transmit this recognition socially when in healthy coral water but not in degraded coral water. However, when we examined cross-species learning, we found that *P. amboinensis* could learn from *P. nagasakiensis* – a rubble-associated congeneric from the same prey guild, in both healthy coral and degraded coral water. This result indicates that alarm cues of *P. nagasakiensis* are not rendered ineffective in degraded coral water contrary to those from *P. amboinensis*. This work raises fascinating questions about the chemistry of alarm cues. We know very little about the chemistry of alarm cues^15^,^41^, but the fact that we see cross-species responses among closely-related species means that animals are likely using similar suites of chemicals^42^,^43^. Whether the failure of *P. ambonensis* to respond to their alarm cues result from deactivation of the cue itself or a failure of the sensory organs is yet to be determined, but our results provide clear interspecific differences in the ability of coral reef species to withstand coral degradation.

Experiments that have simulated near future ocean acidification conditions have found similar results as ours regarding the ‘resilience’ of *P. nagasakiensis* vs. *P. amboinensis* with regards to their alarm cues responses. Raising the CO_2_ level from current day conditions (390 ppm) to 700 or 850 ppm (end of century projection) results in a reduction in the intensity of the response to conspecific alarm cues for *P. nagasakiensis*, but these same elevated CO_2_ levels completely eliminate the responses of *P. amboinensis* to conspecific alarm cues^44^. Other species of damselfish showed intermediate impairment with elevated CO_2_ and hence may be great candidates for future work examining coral degradation. What renders one species’ alarm cues unusable while not affecting the other species’ alarm cues, can likely only be addressed when we gain a thorough understanding of the chemistry of alarm cues.

Our work highlights that the effects of habitat degradation are going to be highly context dependent. *P. amboinensis* settle into a variety of habitats, including live coral, dead coral and rubble at the end of their larval phase at about 15–23 days of age^45^. If they settle into a dead coral habitat, they may suffer from not being able to learn about predators through conditioning with alarm cues. Learning socially provides a great alternative to direct learning with alarm cues, however, given that *P. amboinensis* are unlikely to move into degraded habitats from live coral habitats, the possibility of social learning from conspecifics that themselves learned through alarm cue conditioning, is reduced. However, other heterospecific damselfish, including *P. nagasakiensis*, provide a great source of public information upon which to learn predators. In degraded habitats, the success of ambon damselfish may be enhanced from the transfer of risk-related information rather than impeded through interspecific competition^46^. In addition, information flow between species is known to influence the distribution, both in space and time, of heterospecifics that act as either as information sources or information sinks in the community^47^. If habitat degradation leads to a new directional flow of information between the species adapted to the degraded habitat towards those that are not, it is reasonable to assume a reconfiguration of the community, at least in terms of species sharing the same trophic positions. Future work documenting the change in information connectivity in communities in the face of environmental change should prove fascinating.

## Additional Information

**How to cite this article**: Chivers, D. P. *et al.* Risk assessment and predator learning in a changing world: understanding the impacts of coral reef degradation. *Sci. Rep.* **6**, 32542; doi: 10.1038/srep32542 (2016).
